# 7-Benzyl-3-(4-chloro­phen­yl)-2-isobutyl­amino-5,6,7,8-tetra­hydro­pyrido[4′,3′:4,5]thieno[2,3-*d*]pyrimidin-4(3*H*)-one

**DOI:** 10.1107/S1600536812007246

**Published:** 2012-02-24

**Authors:** Hong Chen, Quan-Bin Liao

**Affiliations:** a Hubei Key Laboratory of Natural Products Research and Development, China Three Gorges University, Yichang 443002, People’s Republic of China; b College of Chemistry and Life Science, China Three Gorges University, Yichang 443002, People’s Republic of China

## Abstract

In the title compound, C_26_H_27_ClN_4_OS, the thienopyrimidine fused-ring system is close to coplanar (r.m.s. deviation = 0.0089 Å), with a maximum deviation of 0.0283 (17) Å for the N atom adjacent to the benzene ring. This ring system forms dihedral angles of 83.51 (3) and 88.20 (5)° with the adjacent benzyl and phenyl rings, respectively. In the crystal, N—H⋯Cl inter­actions and C—H⋯O hydrogen bonds are observed.

## Related literature

For the biological and pharmaceutical properties of compounds containing a fused thienopyrimidine system, see: Amr *et al.* (2010 ▶); Huang *et al.* (2009 ▶); Jennings *et al.* (2005 ▶); Kikuchi *et al.* (2006 ▶); Mavrova *et al.* (2010 ▶); Santagati *et al.* (2002 ▶). For similar crystal structures, see: Xie *et al.* (2008 ▶); Chen *et al.* (2011 ▶).
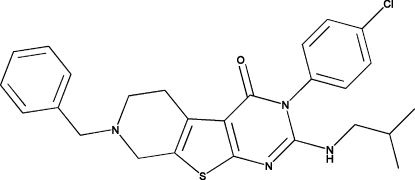


## Experimental

### 

#### Crystal data

C_26_H_27_ClN_4_OS
*M* *_r_* = 479.03
Monoclinic, 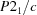

*a* = 17.428 (13) Å
*b* = 9.391 (7) Å
*c* = 16.170 (13) Å
β = 111.995 (7)°
*V* = 2454 (3) Å^3^
*Z* = 4
Mo *K*α radiation
μ = 0.27 mm^−1^
*T* = 296 K
0.26 × 0.24 × 0.20 mm

#### Data collection

Bruker SMART CCD diffractometer
Absorption correction: multi-scan (*SADABS*; Sheldrick, 1996 ▶) *T*_min_ = 0.934, *T*_max_ = 0.949
25485 measured reflections
5611 independent reflections
3883 reflections with *I* > 2σ(*I*)
*R*_int_ = 0.090

#### Refinement

*R*[*F* ^2^ > 2σ(*F* ^2^)] = 0.070
*wR*(*F* ^2^) = 0.164
*S* = 1.06
5611 reflections
300 parameters
H-atom parameters constrained
Δρ_max_ = 0.36 e Å^−3^
Δρ_min_ = −0.26 e Å^−3^

### 

Data collection: *SMART* (Bruker, 1997 ▶); cell refinement: *SAINT* (Bruker, 1997 ▶); data reduction: *SAINT*; program(s) used to solve structure: *SHELXTL* (Sheldrick, 2008 ▶); program(s) used to refine structure: *SHELXTL*; molecular graphics: *SHELXTL*; software used to prepare material for publication: *SHELXTL*.

## Supplementary Material

Crystal structure: contains datablock(s) I, global. DOI: 10.1107/S1600536812007246/nc2265sup1.cif

Structure factors: contains datablock(s) I. DOI: 10.1107/S1600536812007246/nc2265Isup2.hkl

Supplementary material file. DOI: 10.1107/S1600536812007246/nc2265Isup3.cml

Additional supplementary materials:  crystallographic information; 3D view; checkCIF report

## Figures and Tables

**Table 1:** Hydrogen-bond geometry (Å, °)

*D*—H⋯*A*	*D*—H	H⋯*A*	*D*⋯*A*	*D*—H⋯*A*
N4—H4⋯Cl1^i^	0.86	2.73	3.469 (3)	144
C8—H8*B*⋯O1^ii^	0.97	2.59	3.220 (4)	123

Symmetry codes: (i) 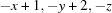; (ii) 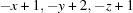.

**Fig. 1.**
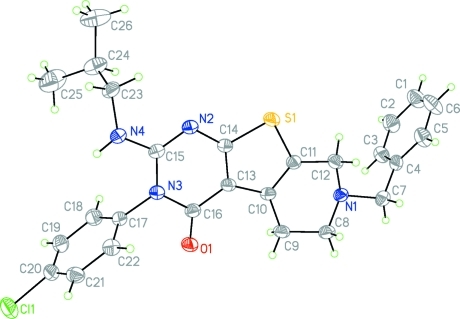
Molecular structure of the title compound with 50% probability displacement ellipsoids.

## supplementary crystallographic information

### Comment

Derivatives of heterocycles containing the thienopyrimidine system have proved to show significant antifungal, antibacterical, anticonvulsant and angiotensin antagonistic activities (Amr *et al.* 2010; Huang *et al.* 2009; Jennings *et al.* 2005; Kikuchi *et al.* 2006; Mavrova *et al.* 2010; Santagati *et al.* 2002). Recently, we have focused on the synthesis of fused heterocyclic systems containing thienopyrimidine *via* aza-wittig reaction under mild conditions. Some X-ray crystal structures of fused pyrimidinone derivatives have been reported (Chen *et al.*, 2011; Xie *et al.*, 2008). The title compound has potential use as a precursor for obtaining bioactive molecules with fluorescence properties. Herein, we report its crystal structure (Fig. 1).

In the crystal structure of the title compound, C_26_H_27_ClN_4_OS, the thienopyrimidine fused-ring system is close to coplanar (r.m.s deviation = 0.0089 Å) with a maximum deviation of 0.0283 (17) Å for atom N(3). This ring system forms dihedral angles of 83.51 (3) and 88.20 (5)° with the adjacent 6-membered rings C1–C6 and C17–C22, respectively. Intermolecular N4—H4···Cl1^i^ interactions, as well as intermolecular hydrogen bonds (C8—H8*B*···O1^ii^), help to stablize the crystal structure (Symmetry codes: (i) -*x* + 1, -*y* + 2, -*z*; (ii) -*x* + 1, -*y* + 2, -*z* + 1) (Table 1).

### Experimental

1-Chloro-4-isocyanatobenzene (2 mmol) under nitrogen atmosphere was added to a solution of iminophosphorane (1.15 g, 2 mmol) in anhydrous dichloromethane (10 ml) at room temperature. When the reaction mixture was left unstirred for 12 h at 273–278 K, iminophosphorane was consumed (TLC monitored). The solvent was removed under reduced pressure and ether/petroleum ether (volume ratio 1:2, 20 ml) was added to precipitate triphenylphosphine oxide. Removal of the solvent gave carbodiimide, which was used directly without further purification. *Iso*-butylamine (2 mmol) was added to the solution of carbodiimide in anhydrous dichloromethane (10 ml). After the reaction mixture was left unstirred for 5–6 h, the solvent was removed and the residual was recrystallized from ethanol and dichloromethane to give the expected title compound in white crystals. Yield: 87%, m.p. 461–462 K. IR (KBr) cm^-1^ 3341 (N—H), 1675 (C═O), 1541, 1433, 1223, 691; ^1^H NMR (CDCl_3_, 600 MHz) δ (p.p.m.): 7.55–7.22 (m, 9H, Ar—H), 4.07 (br, 1H, NH), 3.72 (s, 2H, Ar—CH_2_), 3.60 (s, 2H, NCH_2_-thiophene), 3.18 (t, *J* = 6.3 Hz, 2H, NHCH_2_), 2.98 (t, *J* = 5.7 Hz, 2H, NCH_2_CH_2_), 2.83 (t, *J* = 5.7 Hz, 2H, NCH_2_CH_2_), 1.80–1.76 (m, *J* = 5.4 Hz, 1H, CH), 0.83 (s, 6H, 2 CH_3_); EI–MS (*m/z*, %): 480.06 (*M*+2^+^, 17), 478.01 (*M*^+^, 52), 387.02 (14), 358.96 (57), 303.01 (42), 152.03 (21), 91.09 (100), 44.02 (15). Anal. Calcd. (%) for C_26_H_27_ClN_4_OS: C, 65.19; H, 5.68; N, 11.70. Found (%): C, 65.47; H, 5.85; N, 11.89.

### Refinement

All H atoms were positioned geometrically [C—H = 0.93, 0.96, 0.97 Å and N—H = 0.86 Å] and allowed to ride on their parent atoms, with *U*_iso_(H) = 1.2–1.5*U*_eq_ of the C or N atom.

### Crystal data

**Table:**

C_26_H_27_ClN_4_OS	*F*(000) = 1008
*M**_r_* = 479.03	*D*_x_ = 1.297 Mg m^−^^3^
Monoclinic, *P*2_1_/*c*	Melting point: 462 K
Hall symbol: -P 2ybc	Mo *K*α radiation, λ = 0.71073 Å
*a* = 17.428 (13) Å	Cell parameters from 4592 reflections
*b* = 9.391 (7) Å	θ = 2.5–27.5°
*c* = 16.170 (13) Å	µ = 0.27 mm^−^^1^
β = 111.995 (7)°	*T* = 296 K
*V* = 2454 (3) Å^3^	Prism, colourless
*Z* = 4	0.26 × 0.24 × 0.20 mm

### Data collection

**Table:**

Bruker SMART CCD diffractometer	5611 independent reflections
Radiation source: fine-focus sealed tube	3883 reflections with *I* > 2σ(*I*)
Graphite monochromator	*R*_int_ = 0.090
CCD Profile fitting scans	θ_max_ = 27.5°, θ_min_ = 2.5°
Absorption correction: multi-scan (*SADABS*; Sheldrick, 1996)	*h* = −22→22
*T*_min_ = 0.934, *T*_max_ = 0.949	*k* = −12→12
25485 measured reflections	*l* = −20→20

### Refinement

**Table:**

Refinement on *F*^2^	Primary atom site location: structure-invariant direct methods
Least-squares matrix: full	Secondary atom site location: difference Fourier map
*R*[*F*^2^ > 2σ(*F*^2^)] = 0.070	Hydrogen site location: inferred from neighbouring sites
*wR*(*F*^2^) = 0.164	H-atom parameters constrained
*S* = 1.06	*w* = 1/[σ^2^(*F*_o_^2^) + (0.0535*P*)^2^ + 1.2122*P*] where *P* = (*F*_o_^2^ + 2*F*_c_^2^)/3
5611 reflections	(Δ/σ)_max_ < 0.001
300 parameters	Δρ_max_ = 0.36 e Å^−^^3^
0 restraints	Δρ_min_ = −0.26 e Å^−^^3^

### Special details

**Table:**

Geometry. All e.s.d.'s (except the e.s.d. in the dihedral angle between two l.s. planes) are estimated using the full covariance matrix. The cell e.s.d.'s are taken into account individually in the estimation of e.s.d.'s in distances, angles and torsion angles; correlations between e.s.d.'s in cell parameters are only used when they are defined by crystal symmetry. An approximate (isotropic) treatment of cell e.s.d.'s is used for estimating e.s.d.'s involving l.s. planes.
Refinement. Refinement of *F*^2^ against ALL reflections. The weighted *R*-factor *wR* and goodness of fit *S* are based on *F*^2^, conventional *R*-factors *R* are based on *F*, with *F* set to zero for negative *F*^2^. The threshold expression of *F*^2^ > σ(*F*^2^) is used only for calculating *R*-factors(gt) *etc*. and is not relevant to the choice of reflections for refinement. *R*-factors based on *F*^2^ are statistically about twice as large as those based on *F*, and *R*-factors based on ALL data will be even larger.

### Fractional atomic coordinates and isotropic or equivalent isotropic displacement parameters (Å^2^)

**Table:**

	*x*	*y*	*z*	*U*_iso_*/*U*_eq_	
Cl1	0.63895 (6)	1.09995 (13)	0.09315 (7)	0.0814 (3)	
C1	0.0316 (2)	1.1445 (5)	0.6378 (3)	0.0782 (11)	
H1	−0.0188	1.1831	0.6337	0.094*	
C2	0.0970 (2)	1.2321 (4)	0.6461 (2)	0.0695 (10)	
H2	0.0913	1.3303	0.6480	0.083*	
C3	0.1715 (2)	1.1734 (4)	0.6516 (2)	0.0585 (8)	
H3	0.2158	1.2333	0.6577	0.070*	
C4	0.18193 (19)	1.0276 (3)	0.64841 (19)	0.0482 (7)	
C5	0.1159 (2)	0.9416 (4)	0.6414 (3)	0.0692 (10)	
H5	0.1215	0.8431	0.6404	0.083*	
C6	0.0411 (2)	1.0003 (5)	0.6358 (3)	0.0870 (13)	
H6	−0.0032	0.9408	0.6305	0.104*	
C7	0.26265 (19)	0.9681 (4)	0.65007 (19)	0.0517 (7)	
H7A	0.3077	1.0067	0.7009	0.062*	
H7B	0.2627	0.8654	0.6570	0.062*	
C8	0.35672 (18)	0.9495 (4)	0.57200 (19)	0.0515 (7)	
H8A	0.3558	0.8462	0.5708	0.062*	
H8B	0.3992	0.9794	0.6278	0.062*	
C9	0.37759 (17)	1.0049 (4)	0.49451 (18)	0.0509 (7)	
H9A	0.3894	1.1061	0.5020	0.061*	
H9B	0.4264	0.9567	0.4934	0.061*	
C10	0.30634 (16)	0.9793 (3)	0.40863 (17)	0.0399 (6)	
C11	0.23083 (16)	0.9471 (3)	0.40872 (17)	0.0404 (6)	
C12	0.20962 (17)	0.9400 (3)	0.49018 (18)	0.0470 (7)	
H12A	0.1583	0.9908	0.4794	0.056*	
H12B	0.2016	0.8415	0.5030	0.056*	
C13	0.30607 (15)	0.9815 (3)	0.31940 (17)	0.0394 (6)	
C14	0.23034 (16)	0.9494 (3)	0.25440 (18)	0.0407 (6)	
C15	0.27191 (17)	0.9691 (3)	0.13904 (18)	0.0416 (6)	
C16	0.37323 (17)	1.0126 (3)	0.29188 (18)	0.0429 (6)	
C17	0.41915 (16)	1.0218 (3)	0.16662 (17)	0.0406 (6)	
C18	0.43494 (19)	1.1580 (3)	0.1439 (2)	0.0519 (7)	
H18	0.4005	1.2332	0.1445	0.062*	
C19	0.50245 (19)	1.1807 (4)	0.1202 (2)	0.0549 (8)	
H19	0.5141	1.2717	0.1054	0.066*	
C20	0.55211 (18)	1.0682 (4)	0.11873 (19)	0.0511 (7)	
C21	0.53581 (18)	0.9314 (4)	0.1390 (2)	0.0522 (8)	
H21	0.5690	0.8558	0.1356	0.063*	
C22	0.46907 (18)	0.9086 (3)	0.16437 (19)	0.0474 (7)	
H22	0.4580	0.8177	0.1798	0.057*	
C23	0.17702 (19)	0.9558 (3)	−0.01924 (19)	0.0538 (8)	
H23A	0.1411	0.9049	0.0041	0.065*	
H23B	0.1808	0.9003	−0.0682	0.065*	
C24	0.1383 (2)	1.0970 (4)	−0.0548 (2)	0.0699 (10)	
H24	0.1388	1.1552	−0.0043	0.084*	
C25	0.1863 (3)	1.1749 (5)	−0.1013 (3)	0.1069 (17)	
H25A	0.2408	1.1960	−0.0591	0.160*	
H25B	0.1583	1.2620	−0.1261	0.160*	
H25C	0.1903	1.1162	−0.1481	0.160*	
C26	0.0481 (3)	1.0733 (6)	−0.1165 (3)	0.1126 (18)	
H26A	0.0231	1.1631	−0.1401	0.169*	
H26B	0.0185	1.0301	−0.0835	0.169*	
H26C	0.0461	1.0118	−0.1647	0.169*	
N1	0.27599 (14)	1.0031 (3)	0.56719 (14)	0.0423 (5)	
N2	0.21063 (14)	0.9434 (3)	0.16459 (15)	0.0438 (6)	
N3	0.35216 (13)	0.9995 (3)	0.19807 (14)	0.0419 (5)	
N4	0.25971 (15)	0.9662 (3)	0.05109 (15)	0.0527 (6)	
H4	0.3020	0.9707	0.0360	0.079*	
S1	0.15757 (4)	0.91648 (9)	0.30144 (5)	0.0500 (2)	
O1	0.44421 (12)	1.0478 (3)	0.33911 (13)	0.0578 (6)	

### Atomic displacement parameters (Å^2^)

**Table:**

	*U*^11^	*U*^22^	*U*^33^	*U*^12^	*U*^13^	*U*^23^
Cl1	0.0590 (5)	0.1216 (9)	0.0769 (6)	−0.0133 (5)	0.0407 (5)	−0.0021 (6)
C1	0.064 (2)	0.086 (3)	0.100 (3)	0.006 (2)	0.048 (2)	0.002 (2)
C2	0.071 (2)	0.064 (2)	0.084 (3)	0.0060 (18)	0.042 (2)	−0.0009 (19)
C3	0.0549 (19)	0.060 (2)	0.064 (2)	−0.0091 (15)	0.0263 (17)	−0.0018 (16)
C4	0.0508 (17)	0.059 (2)	0.0379 (15)	−0.0035 (14)	0.0198 (14)	0.0015 (13)
C5	0.070 (2)	0.062 (2)	0.084 (3)	−0.0081 (18)	0.039 (2)	0.0008 (18)
C6	0.062 (2)	0.086 (3)	0.126 (4)	−0.018 (2)	0.051 (3)	−0.003 (3)
C7	0.0520 (18)	0.062 (2)	0.0397 (16)	0.0025 (14)	0.0156 (14)	0.0039 (13)
C8	0.0393 (16)	0.070 (2)	0.0397 (15)	0.0056 (14)	0.0086 (13)	0.0014 (14)
C9	0.0308 (14)	0.079 (2)	0.0394 (15)	0.0008 (14)	0.0087 (12)	−0.0056 (14)
C10	0.0313 (13)	0.0452 (16)	0.0382 (14)	0.0002 (11)	0.0074 (11)	−0.0034 (11)
C11	0.0371 (14)	0.0460 (17)	0.0352 (13)	−0.0030 (11)	0.0102 (12)	−0.0041 (11)
C12	0.0397 (15)	0.0570 (19)	0.0441 (16)	−0.0074 (13)	0.0154 (13)	−0.0044 (13)
C13	0.0301 (13)	0.0477 (17)	0.0371 (14)	−0.0011 (11)	0.0087 (11)	−0.0014 (11)
C14	0.0328 (14)	0.0461 (17)	0.0394 (14)	−0.0035 (11)	0.0091 (12)	−0.0007 (11)
C15	0.0379 (14)	0.0441 (17)	0.0376 (14)	0.0008 (11)	0.0082 (12)	0.0002 (11)
C16	0.0364 (14)	0.0502 (18)	0.0376 (14)	0.0011 (12)	0.0087 (12)	−0.0021 (12)
C17	0.0358 (14)	0.0475 (17)	0.0365 (14)	−0.0018 (12)	0.0111 (12)	0.0013 (11)
C18	0.0524 (18)	0.0488 (19)	0.0556 (18)	0.0061 (14)	0.0216 (15)	0.0053 (14)
C19	0.0542 (18)	0.056 (2)	0.0566 (18)	−0.0067 (15)	0.0230 (16)	0.0079 (14)
C20	0.0418 (16)	0.070 (2)	0.0422 (16)	−0.0064 (14)	0.0162 (14)	−0.0015 (14)
C21	0.0429 (16)	0.063 (2)	0.0484 (17)	0.0054 (14)	0.0140 (14)	−0.0098 (14)
C22	0.0459 (16)	0.0445 (17)	0.0498 (16)	−0.0005 (13)	0.0158 (14)	−0.0005 (13)
C23	0.0480 (17)	0.066 (2)	0.0369 (15)	−0.0093 (14)	0.0037 (14)	0.0019 (13)
C24	0.065 (2)	0.066 (2)	0.059 (2)	0.0075 (18)	0.0009 (18)	−0.0100 (17)
C25	0.109 (4)	0.074 (3)	0.102 (3)	−0.022 (3)	−0.001 (3)	0.032 (3)
C26	0.068 (3)	0.124 (4)	0.104 (4)	0.014 (3)	−0.015 (3)	0.004 (3)
N1	0.0377 (12)	0.0533 (15)	0.0338 (11)	0.0022 (10)	0.0107 (10)	−0.0011 (10)
N2	0.0344 (12)	0.0553 (16)	0.0363 (12)	−0.0041 (10)	0.0071 (10)	−0.0018 (10)
N3	0.0338 (12)	0.0507 (15)	0.0380 (12)	−0.0038 (10)	0.0098 (10)	0.0003 (10)
N4	0.0385 (13)	0.0814 (19)	0.0333 (12)	−0.0024 (12)	0.0079 (11)	0.0015 (11)
S1	0.0332 (4)	0.0741 (6)	0.0398 (4)	−0.0117 (3)	0.0102 (3)	−0.0071 (3)
O1	0.0337 (10)	0.0945 (18)	0.0403 (11)	−0.0104 (10)	0.0081 (9)	−0.0057 (10)

### Geometric parameters (Å, º)

**Table:**

Cl1—C20	1.738 (3)	C14—N2	1.362 (4)
C1—C6	1.366 (6)	C14—S1	1.734 (3)
C1—C2	1.371 (5)	C15—N2	1.304 (3)
C1—H1	0.9300	C15—N4	1.357 (4)
C2—C3	1.382 (5)	C15—N3	1.396 (3)
C2—H2	0.9300	C16—O1	1.231 (3)
C3—C4	1.384 (5)	C16—N3	1.426 (4)
C3—H3	0.9300	C17—C22	1.383 (4)
C4—C5	1.375 (5)	C17—C18	1.386 (4)
C4—C7	1.505 (4)	C17—N3	1.453 (3)
C5—C6	1.388 (5)	C18—C19	1.383 (4)
C5—H5	0.9300	C18—H18	0.9300
C6—H6	0.9300	C19—C20	1.372 (4)
C7—N1	1.481 (4)	C19—H19	0.9300
C7—H7A	0.9700	C20—C21	1.381 (4)
C7—H7B	0.9700	C21—C22	1.387 (4)
C8—N1	1.469 (4)	C21—H21	0.9300
C8—C9	1.521 (4)	C22—H22	0.9300
C8—H8A	0.9700	C23—N4	1.467 (4)
C8—H8B	0.9700	C23—C24	1.501 (5)
C9—C10	1.495 (4)	C23—H23A	0.9700
C9—H9A	0.9700	C23—H23B	0.9700
C9—H9B	0.9700	C24—C25	1.507 (6)
C10—C11	1.351 (4)	C24—C26	1.530 (5)
C10—C13	1.441 (4)	C24—H24	0.9800
C11—C12	1.497 (4)	C25—H25A	0.9600
C11—S1	1.750 (3)	C25—H25B	0.9600
C12—N1	1.470 (3)	C25—H25C	0.9600
C12—H12A	0.9700	C26—H26A	0.9600
C12—H12B	0.9700	C26—H26B	0.9600
C13—C14	1.378 (4)	C26—H26C	0.9600
C13—C16	1.429 (4)	N4—H4	0.8600
			
C6—C1—C2	119.6 (4)	O1—C16—N3	118.8 (3)
C6—C1—H1	120.2	O1—C16—C13	127.7 (3)
C2—C1—H1	120.2	N3—C16—C13	113.5 (2)
C1—C2—C3	119.6 (4)	C22—C17—C18	120.8 (3)
C1—C2—H2	120.2	C22—C17—N3	119.6 (3)
C3—C2—H2	120.2	C18—C17—N3	119.4 (3)
C2—C3—C4	121.7 (3)	C19—C18—C17	119.3 (3)
C2—C3—H3	119.2	C19—C18—H18	120.3
C4—C3—H3	119.2	C17—C18—H18	120.3
C5—C4—C3	117.8 (3)	C20—C19—C18	119.6 (3)
C5—C4—C7	122.1 (3)	C20—C19—H19	120.2
C3—C4—C7	120.1 (3)	C18—C19—H19	120.2
C4—C5—C6	120.6 (4)	C19—C20—C21	121.7 (3)
C4—C5—H5	119.7	C19—C20—Cl1	118.9 (3)
C6—C5—H5	119.7	C21—C20—Cl1	119.4 (2)
C1—C6—C5	120.7 (4)	C20—C21—C22	118.9 (3)
C1—C6—H6	119.6	C20—C21—H21	120.6
C5—C6—H6	119.6	C22—C21—H21	120.6
N1—C7—C4	111.3 (2)	C17—C22—C21	119.7 (3)
N1—C7—H7A	109.4	C17—C22—H22	120.2
C4—C7—H7A	109.4	C21—C22—H22	120.2
N1—C7—H7B	109.4	N4—C23—C24	114.1 (3)
C4—C7—H7B	109.4	N4—C23—H23A	108.7
H7A—C7—H7B	108.0	C24—C23—H23A	108.7
N1—C8—C9	111.0 (2)	N4—C23—H23B	108.7
N1—C8—H8A	109.4	C24—C23—H23B	108.7
C9—C8—H8A	109.4	H23A—C23—H23B	107.6
N1—C8—H8B	109.4	C23—C24—C25	111.4 (3)
C9—C8—H8B	109.4	C23—C24—C26	109.0 (3)
H8A—C8—H8B	108.0	C25—C24—C26	111.8 (4)
C10—C9—C8	109.8 (2)	C23—C24—H24	108.2
C10—C9—H9A	109.7	C25—C24—H24	108.2
C8—C9—H9A	109.7	C26—C24—H24	108.2
C10—C9—H9B	109.7	C24—C25—H25A	109.5
C8—C9—H9B	109.7	C24—C25—H25B	109.5
H9A—C9—H9B	108.2	H25A—C25—H25B	109.5
C11—C10—C13	111.5 (2)	C24—C25—H25C	109.5
C11—C10—C9	120.4 (2)	H25A—C25—H25C	109.5
C13—C10—C9	128.1 (2)	H25B—C25—H25C	109.5
C10—C11—C12	124.9 (2)	C24—C26—H26A	109.5
C10—C11—S1	112.7 (2)	C24—C26—H26B	109.5
C12—C11—S1	122.4 (2)	H26A—C26—H26B	109.5
N1—C12—C11	110.6 (2)	C24—C26—H26C	109.5
N1—C12—H12A	109.5	H26A—C26—H26C	109.5
C11—C12—H12A	109.5	H26B—C26—H26C	109.5
N1—C12—H12B	109.5	C12—N1—C8	109.9 (2)
C11—C12—H12B	109.5	C12—N1—C7	109.7 (2)
H12A—C12—H12B	108.1	C8—N1—C7	110.4 (2)
C14—C13—C16	118.0 (2)	C15—N2—C14	114.9 (2)
C14—C13—C10	113.8 (2)	C15—N3—C16	122.5 (2)
C16—C13—C10	128.2 (2)	C15—N3—C17	121.4 (2)
N2—C14—C13	127.5 (3)	C16—N3—C17	116.1 (2)
N2—C14—S1	121.8 (2)	C15—N4—C23	122.5 (3)
C13—C14—S1	110.7 (2)	C15—N4—H4	118.8
N2—C15—N4	120.2 (2)	C23—N4—H4	118.8
N2—C15—N3	123.4 (2)	C14—S1—C11	91.35 (14)
N4—C15—N3	116.4 (2)		
			
C6—C1—C2—C3	0.4 (6)	C19—C20—C21—C22	−2.2 (5)
C1—C2—C3—C4	0.5 (5)	Cl1—C20—C21—C22	176.3 (2)
C2—C3—C4—C5	−1.4 (5)	C18—C17—C22—C21	0.0 (4)
C2—C3—C4—C7	177.0 (3)	N3—C17—C22—C21	−176.5 (3)
C3—C4—C5—C6	1.4 (5)	C20—C21—C22—C17	1.7 (4)
C7—C4—C5—C6	−176.9 (4)	N4—C23—C24—C25	64.8 (4)
C2—C1—C6—C5	−0.4 (7)	N4—C23—C24—C26	−171.4 (3)
C4—C5—C6—C1	−0.5 (7)	C11—C12—N1—C8	−46.5 (3)
C5—C4—C7—N1	110.2 (3)	C11—C12—N1—C7	−168.0 (2)
C3—C4—C7—N1	−68.1 (4)	C9—C8—N1—C12	68.1 (3)
N1—C8—C9—C10	−50.3 (3)	C9—C8—N1—C7	−170.8 (3)
C8—C9—C10—C11	15.6 (4)	C4—C7—N1—C12	−61.3 (3)
C8—C9—C10—C13	−163.2 (3)	C4—C7—N1—C8	177.5 (3)
C13—C10—C11—C12	−178.3 (3)	N4—C15—N2—C14	−180.0 (3)
C9—C10—C11—C12	2.7 (4)	N3—C15—N2—C14	0.5 (4)
C13—C10—C11—S1	0.7 (3)	C13—C14—N2—C15	1.3 (4)
C9—C10—C11—S1	−178.3 (2)	S1—C14—N2—C15	−179.3 (2)
C10—C11—C12—N1	12.6 (4)	N2—C15—N3—C16	−3.5 (4)
S1—C11—C12—N1	−166.3 (2)	N4—C15—N3—C16	177.0 (3)
C11—C10—C13—C14	−0.7 (4)	N2—C15—N3—C17	177.5 (3)
C9—C10—C13—C14	178.2 (3)	N4—C15—N3—C17	−2.0 (4)
C11—C10—C13—C16	179.2 (3)	O1—C16—N3—C15	−175.8 (3)
C9—C10—C13—C16	−1.9 (5)	C13—C16—N3—C15	4.3 (4)
C16—C13—C14—N2	−0.1 (5)	O1—C16—N3—C17	3.3 (4)
C10—C13—C14—N2	179.8 (3)	C13—C16—N3—C17	−176.6 (2)
C16—C13—C14—S1	−179.6 (2)	C22—C17—N3—C15	−93.6 (3)
C10—C13—C14—S1	0.3 (3)	C18—C17—N3—C15	89.9 (3)
C14—C13—C16—O1	177.5 (3)	C22—C17—N3—C16	87.3 (3)
C10—C13—C16—O1	−2.3 (5)	C18—C17—N3—C16	−89.2 (3)
C14—C13—C16—N3	−2.6 (4)	N2—C15—N4—C23	9.3 (4)
C10—C13—C16—N3	177.6 (3)	N3—C15—N4—C23	−171.2 (3)
C22—C17—C18—C19	−1.2 (4)	C24—C23—N4—C15	92.6 (4)
N3—C17—C18—C19	175.3 (3)	N2—C14—S1—C11	−179.4 (2)
C17—C18—C19—C20	0.7 (5)	C13—C14—S1—C11	0.1 (2)
C18—C19—C20—C21	1.0 (5)	C10—C11—S1—C14	−0.5 (2)
C18—C19—C20—Cl1	−177.5 (2)	C12—C11—S1—C14	178.5 (3)

### Hydrogen-bond geometry (Å, º)

**Table:**

*D*—H···*A*	*D*—H	H···*A*	*D*···*A*	*D*—H···*A*
N4—H4···Cl1^i^	0.86	2.73	3.469 (3)	144
C8—H8*B*···O1^ii^	0.97	2.59	3.220 (4)	123

Symmetry codes: (i) −*x*+1, −*y*+2, −*z*; (ii) −*x*+1, −*y*+2, −*z*+1.
